# *In Vitro* Antimicrobial and Antiproliferative Activity of *Amphipterygium adstringens*

**DOI:** 10.1155/2015/175497

**Published:** 2015-09-14

**Authors:** A. Rodriguez-Garcia, I. T. A. Peixoto, M. J. Verde-Star, S. De la Torre-Zavala, H. Aviles-Arnaut, A. L. T. G. Ruiz

**Affiliations:** ^1^Institute of Biotechnology, Faculty of Biological Sciences, Autonomous University of Nuevo Leon, Avenida Universidad S/N, Ciudad Universitaria, CP 66455, San Nicolas de los Garza, NL, Mexico; ^2^Escola Bahiana de Medicina e Saúde Pública (EBMSP), Avenida Silveira Martins 3386, 41150 100 Salvador, BA, Brazil; ^3^Chemical, Biological and Agricultural Pluridisciplinary Research Center (CPQBA), University of Campinas (UNICAMP), CP 6171, 13083-970 Campinas, SP, Brazil

## Abstract

*Amphipterygium adstringens* is a plant widely used in Mexican traditional medicine for its known anti-inflammatory and antiulcer properties. In this work, we evaluated the *in vitro* antimicrobial and antiproliferative activities of the methanolic extract of *A. adstringens* against oral pathogens such as *Streptococcus mutans*, *Porphyromonas gingivalis*, *Aggregatibacter actinomycetemcomitans*, *Candida albicans*, and *Candida dubliniensis*, using microdilution (MIC) and agar diffusion methods (MBC), and the antiproliferative activity evaluating total growth inhibition (TGI) by staining the protein content with sulforhodamine B (SRB), using nine human cancer cell lines. Crude extract (CE) of *A. adstringens* showed some degree of activity against one or more of the strains with a MIC from 0.125 mg/mL to 63 mg/mL and MBC from 1.6 to 6.3 mg/mL and cytotoxic activity, particularly against NCI-ADR/RES, an ovarian cell line expressing multiple resistance drugs phenotype. The CE is a complex mixture of possible multitarget metabolites that could be responsible for both antimicrobial and antiproliferative activities, and further investigation is required to elucidate the identity of active compounds. Nevertheless the CE itself is useful in the development of new antimicrobial treatment based on natural products to prevent oral diseases and as alternative natural source for cancer treatment and prevention.

## 1. Introduction

Natural molecules and products reemerge as promising sources of complex multitarget mixtures that are used as alternative therapeutic agents for various conditions, including infections and chronic diseases [1, 2], such as oral diseases [3–5] or digestive cancers [6].

**Figure 1:**
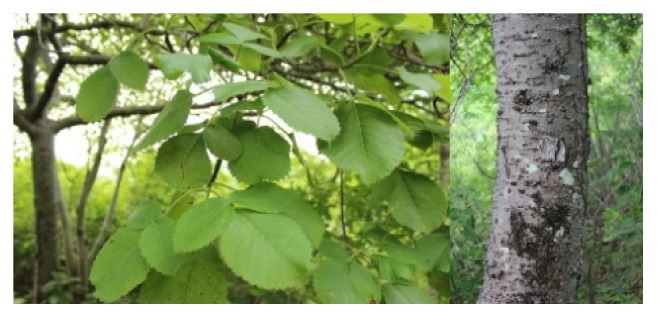
Whole plant and bark of Amphipterygium adstringens growing in Michoacan, Mexico.

*Amphipterygium adstringens* Schiede ex Schlecht (Julianaceae) is an endemic species in Mexico commonly known as “cuachalalate.”* A. adstringens* is a dioecious tree of approximately 5–10 meters in height and 40 cm in diameter, with the twisted trunk, gray bark with large scales, and rounded lenticels exposed and oblong leaves from 6 to 15 cm (Figure 1), clustered at the tips of branches in numbers from 3 to 5 [7]. Its location is in the Mexican Pacific coastline from Nayarit to Oaxaca, and other states of Mexico such as Puebla and Morelos [8].

The bark has traditionally been used by healers to treat gastritis, gastric ulcers [9], gastrointestinal cancer [10, 11], colic, fever [12, 13], and also tooth pain [14]. Moreover, anti-inflammatory [15], hypocholesterolemic [16], antifungal [17], and antiprotozoal [18] activities have been reported as properties for this plant. Recent studies have shown that the active component responsible for the plant properties is the anacardic acid [19] which exerts antioxidant, anti-inflammatory [20], antitumoral [21], antiulcer and antimicrobial activities [9, 19].

A wide range of uses and diversity of biological activities reported reminds us of a complex mixture of multitarget compounds, which is a common characteristic in several medicinal plants and similar to others [22].

Microbial communities in the mouth have been shown to cause infectious diseases such as dental caries [23], candidiasis [24], gingivitis and periodontitis [25], and other chronic or systemic diseases [26–28].

*Streptococcus mutans* is the main pathogenic agent related to the initiation of dental caries. Its virulence relies on its ability to form biofilms on teeth surface, to degrade carbohydrates (particularly refined sugars from food) with the formation of large amounts of organic acids that demineralize tooth enamel, and to adapt and tolerate environmental stresses, particularly low pH (aciduricity) [29].

*Porphyromonas gingivalis* and* Aggregatibacter actinomycetemcomitans* are bacteria often isolated from the subgingival biofilm [30, 31] and associated with periodontitis and gingivitis. Dental biofilm stimulates the release of proteolytic enzymes, which cause disruption of the epithelial junction, gingival and alveolar bone loss, increasing tooth mobility, and, ultimately, tooth loss [32]. Dental biofilm induce a peripheral neutrophil response [33] and it has been associated with systemic conditions such as infective endocarditis [34], cardiovascular disease [35], preterm and low birth weight [36], and pancreatic cancer [37]. Additionally, the constantly observed correlation between oral microbiome (especially biofilm builders) [28] with chronic inflammatory disease and cancer triggers the need for further research that contributes with novel sources of bioactive compounds.

Oropharyngeal candidiasis, caused by* Candida*, is a local infection commonly seen in infants, older adults who wear dentures, patients treated with antibiotics, chemotherapy, or radiation therapy on head and neck, and those with cellular immune deficiency states, such as HIV infection [38]. Moreover,* Candida* species are the fourth leading cause of healthcare-associated infections, accounting for approximately 11% of all infections;* Candida* spp. are also responsible for nearly 12% of all central line-associated bloodstream infections, preceded only by* Staphylococcus aureus* and* Enterococcus* species [39].* Candida albicans* is often guilty in oral candidiasis and has been implicated in persistent apical periodontitis [40]. On the other side,* Candida dubliniensis* was involved with denture stomatitis and oropharyngeal candidiasis in immunocompromised subject [41, 42] and has an invasive history in survival of head or neck cancers patients [43]. Treatments for these infections have grown to be a great challenge, because of a rise in the frequency of infections and an increasing resistance to standard antifungal therapy.

The increasing cancer incidence is closely related to the raise in life expectancy and longevity that consequently results in a longer exposure to carcinogenic agents such as pollution, UV radiation, and microorganisms [44]. The reduction of pathogens associated with systemic and oral infections, using natural products to inhibit the ability to form biofilms, could be an effective approach to prevent and control oral diseases.

Moreover, there has been a decline in research, development, and approvals of new antimicrobial agents. For example, from 1983 to 1987 compared to from 1998 to 2002, there was a 56% decrease in new antibacterial agents approved by the FDA (Food and Drugs Administration). Among other reasons, one main cause is the high cost with a low rate of return compared to drugs used to treat chronic conditions, limitations on the use of newly approved antibacterial agents, and trial design and regulatory issues [45]. This engages the scientific community to continue obtaining research-based information towards the application and use of traditional medicine that medical health care systems can rely on.

With this approach, we studied the antimicrobial activity of CE of* A. adstringens* against microorganisms associated with oral infections, and the possible antiproliferative effect against a well-defined panel of human cancer cells lines. This is, undoubtedly, a significant contribution in the field of alternative medicine towards the elaboration of novel antimicrobial agents to prevent and treat oral infections and to reduce the potential risk of systemic chronic diseases and cancer.

## 2. Materials and Methods

### 2.1. Plant Material

*Amphipterygium adstringens* bark was obtained by Dr. Julia Verde from the Department of Chemistry, Faculty of Biological Sciences, Autonomous University of Nuevo Leon (UANL). The taxonomic identification was made by Dr. Mauricio Gonzalez, following the last revision of the genus* Amphipterygium* (Julianaceae) [46]. A voucher specimen was deposited as IB1 in the Institute of Biotechnology, Faculty of Biological Sciences, UANL.

### 2.2. Preparation of Plant Extracts

The bark of* A. adstringens* was air-dried at 45°C and ground in a Wiley type mill to a fine powder. About 390 g of powder was macerated with 500 mL methanol by shaking at 100 rpm for 72 hours. The extract was filtered with filter paper (Whatman Grade 1) to remove debris and filter-sterilized with 0.20 *μ*m syringe filter (Corning, NY). Extract was concentrated using a rotary evaporator (Yamato RE801), under reduced pressure at 30°C, providing 52.7 g of crude extract. The crude extract was stored at 4°C until its use.

### 2.3. Determination of Antimicrobial Activity

The antimicrobial effect of the crude extract of* A. adstringens* was determined in a preliminary study using the Kirby-Bauer disk diffusion method [47] against several human pathogens (data not shown). This scrutiny preceded the experimental design to determine MIC and MBC/MFC of* A. adstringens* crude extract against oral pathogens.

### 2.4. Microbial Growth Conditions

Strains were cultured following their specific requirements.* S. mutans* (ATCC 700610) was cultured on BHI and 5% CO_2_.* P. gingivalis* (ATCC 33277) and* A. actinomycetemcomitans* (ATCC 43718) was cultured on TSB (Tryptic Soy Broth) and carried out in anaerobic conditions.* Escherichia coli* (ATCC 43895) and* Escherichia coli* (ATCC 0157-H7) were cultured on TSB in aerobic atmosphere and were used as biological control to validate microbicidal activity assay due to their multidrug resistance.* Candida albicans* (CBS-562) and* Candida dubliniensis* (CBS-7987) were cultured on Sabouraud dextrose agar in aerobic conditions [48]. All strains were incubated at 35°C for 48 hours. Chlorhexidine gluconate 0.12% (Sigma-Aldrich, St. Louis, MO, USA) was used as positive control for bactericidal activity due to its antibacterial spectrum against Gram-positive and Gram-negative bacteria and actual application on oral hygiene; Fluconazole (Pfizer), an oral azole, was selected as positive control against* Candida* because it is frequently used in patients who suffer oropharyngeal and esophageal candidiasis.

### 2.5. Minimum Inhibitory Concentration (MIC)

The minimum inhibitory concentrations (MIC) were determined by microdilution broth method according to the Clinical and Laboratory Standards Institute [49], using 96-well plates. The microbial inoculum was prepared and adjusted to 5 × 10^5^ colony forming units (CFU)/mL. The CE was dissolved in 1% DMSO followed by dilution in tryptic soy broth, brain heart infusion, or Sabouraud (depending on the strain), and, then, serial twofold to threefold dilutions were made in a concentration ranged from 78 to 0.01 mg/mL, in sterile water. 96-well plates were incubated at 35°C/24 hours aerobically and anaerobically. MIC was determined as the lowest concentration of CE that inhibited microorganism growth. The experiment was performed by duplicate in three independent tests.

### 2.6. Minimum Bactericidal Concentration (MBC) and Minimum Fungicidal Concentration (MFC)

The MBC or MFC was determined removing the samples from the wells of the microplate with no visible bacterial growth and subcultured in agar (TBS, BHI, or Sabouraud), depending on the strain. The lowest concentration of the subculture with no growth was considered the minimum bactericidal concentration (MBC) or the minimum fungicidal concentration (MFC).

### 2.7. Statistical Analysis

Results were expressed as mean ± SD for analysis performed in triplicate. Statistical analysis of the data was performed by Analysis of Variance (ANOVA) followed by Duncan test using SPSS 17.0 software. Probability values of *P* ≤ 0.05 were considered to be significant.

### 2.8. Human Cell Lines Panel

Human tumor cell lines U251 (CNS), UACC-62 (skin), and OVCAR (ovary); NCI-ADR/RES (ovary multidrug resistant phenotype); 786-O (kidney); NCI-H460 (non-small-cell lung); PC-3 (prostate); HT-29 (colon); and K562 (bone marrow) were kindly provided by M. A. Frederick (National Cancer Institute, USA). Stock culture was grown in 25 cm^3^ flasks cell cultures (Nunc) with 5 mL of RPMI-1640 (Gibco-BRL, Gran Island, NY, USA) supplemented with 5% fetal bovine serum (FBS, Gibco-BRL, Gran Island, NY, USA) plus penicillin/streptomycin (1000 *μ*g/mL : 1000 IU/mL) and incubated at 37°C in humid atmosphere with 5% CO_2_. The cell line has less than 20 passages as laboratory good practices.

### 2.9. Antiproliferative Activity

*In vitro* antiproliferative activity was performed using preincubated (24 hours at 37°C) cell lines into 96-well plates (Nunc), which were exposed at 0.25, 2.5, 25, and 250 *μ*g/mL of* A. adstringens* CE for 48 hours. Cells were fixed with 50% trichloroacetic acid (30 min at 4°C) and cell proliferation was determined by spectrophotometric quantification (540 nm) of cellular protein content using sulforhodamine B assay. Using the concentration-response curve for each cell line, total growth inhibition (TGI) was defined as the concentration value that produces total growth inhibition and was determined by nonlinear regression analysis using the software Origin 7.5 (OringinLab Corporation, Northampton MA, USA). This value corresponded to the test extract concentration necessary to inhibit proliferation of the cells. Doxorubicin was used as positive control. The assay was performed by triplicate per concentration of CE in three independent tests.

## 3. Results

### 3.1. Antimicrobial Activity

Methanolic extract of* A. adstringens* used in this work showed antimicrobial activity against all microbial strains tested with concentrations between 0.125 and 63 mg/mL (Table 1). The most sensitive strain was* S. mutans* with a MIC value of 0.125 mg/mL and MBC at 0.31 mg/mL.* Candida* strains showed a MIC and MFC of 0.4 mg/mL and 1.6 mg/mL, respectively.* P. gingivalis* and* Escherichia coli* 43895 showed MIC/MBC values of 37 mg/mL. Similar results are exhibited by* A. actinomycetemcomitans* and* Escherichia coli* O157:H7 being inhibited at 63 mg/mL. Pure chlorhexidine (positive control) showed a MIC of 0.04 mg/mL and a MBC of 2.4 mg/mL, and 0.06 mg/mL and 1.2 mg/mL for Fluconazole.

### 3.2. Antiproliferative Activity

In this model,* in vitro* cytotoxicity determination of the CE of* A. adstringens* demonstrated total inhibition of tumor cell growth at concentrations ranging from 7.30 to 27.50 *μ*g/mL (Table 2) and showing the most potent activity against ovary adenocarcinoma (4 *μ*g/mL).

## 4. Discussion

There exists an increasing prevalence of infections worldwide, due to, among other reasons, the growing resistance of pathogens to antibiotics and antifungals as well as alterations in the autoimmune system. Diseases as caries, periodontitis and gingivitis, oropharyngeal candidiasis, and cancer have had profound effects on human health. Microbial species in oral cavity are commensals under normal physiological conditions. The host immune response, supplemented by excellent oral hygiene, is sufficient to maintain healthy tissues. However, failures in immune defense or plaque control result in development of gingival inflammation [25–27].

In this investigation, we tested the crude extract of* A. adstringens*, against oral pathogens and human cancer cell lines and, as expected, we unambiguously demonstrated that the methanolic extract exerts significant antimicrobial and antiproliferative activities. Additionally our results provide novel evidence that* A. adstringens* also exerts growth control against* Candida*, and these results represent the first report for this plant.

In regard of activity shown against* S. mutans,* our results showed that inhibitory concentrations of* A. adstringens* CE reached 0.125 mg/mL (MIC) and 0.31 mg/mL (MBC), while chlorhexidine had MIC and MBC values of 0.4 and 2.4 mg/mL, respectively. On the other hand, biocidal activity against periodontopathogens showed that the crude extract of* A. adstringens* had a MIC and MBC of 37 mg/mL for* P. gingivalis* and 63 mg/mL for* A. actinomycetemcomitans.* These results are consistent with previous work demonstrating higher sensitivity of* S. mutans* than periodontopathogens to medicinal plant components [19, 50, 51].

Chlorhexidine is the most common mouthwash product used for caries and periodontal diseases and kills bacteria by causing a precipitation or coagulation of the cytoplasmic content. It is an effective antiplaque agent developed to date, but it has been proven that prolonged use causes several undesirable side effects; besides many clinical trials have shown that taste of chlorhexidine is not well accepted by children [52]. Our results, along with previous reports of* A. adstringens*, enhance proposals to include natural remedies as components of mouthwash, toothpaste, and delivery systems [53] to control dental plaque.

As for activity of* A. adstringens* CE against yeasts, our findings indicated a strong antifungal activity showing MIC and MBC values of 0.4 mg/mL and MFC of 1.6 mg/mL. Those are the lowest among all MIC values shown in our data, suggesting that* A. adstringens* components exert fungicidal activity against candidiasis causal agents. By far, this represents our most original and promising evidence, considering the continuous quest for new and improved products, with emphasis placed on natural/nature identical compounds with the fewest side effects. Fluconazole is commonly used as therapy for candidiasis in HIV and cancer patients, but the rise in prevalence of fungal infections and drug resistance has exacerbated the need of novel antifungal compounds. Our results provide evidence of potential antifungal application of* A. adstringens* offering an alternative therapy to fluconazole.

As mentioned before, oral microbiota diversity is closely related to health conditions and systemic diseases such as cardiovascular and inflammatory diseases and cancer. This is why we designed experiments to holistically test both biological activity against oral pathogens and antiproliferative activity on human cancer cell lines.

The model used in this work to evaluate the antiproliferative activity was the assay with Sulforhodamine B (an anionic dye) that allowed the evaluation of antitumor activity through exposure of selected human tumor cell lines during exponential growth phase, at different concentrations of the* A. adstringens* extract determining total growth inhibition (TGI) [54].

Antiproliferative activity of the CE showed lowest TGI results on ovarian cancer OVCAR-3 adenocarcinoma with a TGI = 4.4 *μ*g/mL, followed by melanoma UACC-62 and colon adenocarcinoma II HT-29 with TGI = 7.3 and 7.9 *μ*g/mL, respectively. These are significant activities at low concentrations considering that CE is a mixture of nonpurified compounds, such as those found in actual traditional use of* A. adstringens.* Consistent with our results, other authors reported* A. adstringens* activity with TGI = 20.4 *μ*g/mL to 48.2 *μ*g/mL and 27.4 *μ*g/mL to 38.3 *μ*g/mL in the cell lines U251 and PC-3, respectively [10]. Similarly, low TGI values for CE were proved in every tumor cell line included in the selected panel.

Interestingly, NCI/ADR-RES ovarian tumor cell line, expressing high levels of MDR1 and P-glycoprotein, which are related to multidrug resistance [55, 56], showed best TGI values (26.40 *μ*g/mL), whereas doxorubicin showed almost no effect at concentrations of 25 *μ*g/mL.

This data confirms that complex mixtures of plants, essential oils, and natural alternative therapeutics that have been widely used in traditional medicine represent an effective, safe, and evidence based treatment for acute and chronic infections as well as chronic health conditions. Preparations and formulations of this CE can be improved and applied for daily use in dental products to prevent caries and periodontal diseases and concomitantly weaken the risk of cancer.

## 5. Conclusions

The findings of the present study demonstrated a broad potential using the heterogeneous mixture of CE of* A. adstringens,* where the synergy of all components could be acting against all the strains tested, especially against* S. mutans* and* Candida* spp.* A. adstringens* has shown selective antiproliferative activity against multidrug resistant cell line NCI/ADR-RES. These effective antimicrobial and antiproliferative activities make* A. adstringens* a good candidate for further development of antimicrobial products for oral infectious therapy and cancer prevention and treatment. However, further research should be made to test whether secondary metabolites from* A. adstringens* maintain reported bioactivity when separated or act as a synergistic complex mixture of phytochemicals.

## Figures and Tables

**Table 1:** Antimicrobial activity of the crude extract of *A. adstringens*.

Pathogens	Reference	MIC^*∗*^(mg/mL)	MBC^*∗∗*^/MFC^*∗∗∗*^(mg/mL)
*Streptococcus mutans*	ATCC 700610	0.125	0.31^a^
*Porphyromonas gingivalis*	ATCC 33277	37	37^c^
*Aggregatibacter actinomycetemcomitans*	ATCC 43718	63	63^c^
*Candida albicans*	CBS-562	0.4	1.6^b^^*∗∗∗*^
*Candida dubliniensis*	CBS-7987	0.4	1.6^b^^*∗∗∗*^
*Escherichia coli*	ATCC 43895	37	37^c^
*Escherichia coli H7*	ATCC 0157:H7	63	63^c^
Chlorhexidine	SIGMA	0.04	2.4
Fluconazole	SIGMA	0.06	1.2

The results represent the means ± SD from three independent experiments. Different letter represents statistically significant differences among pathogens groups as determined by one-way ANOVA and Duncan test. Chlorhexidine and Fluconazole are controls.

^*∗*^MIC: minimum inhibitory concentration.

^*∗∗*^MBC: minimum bactericidal concentration.

^*∗∗∗*^MFC: minimum fungicidal concentration.

**Table 2:** Cell lines used in the evaluation of antiproliferative activity.

Cell line	Organ/disease	Origin tissue	Total growth inhibition (*µ*g/mL)	
			CE	DOX
U251	CNS; glioma	Ectoderm	26.10	<0.025
UACC-62	Skin; melanoma	Ectoderm	7.30	0.028
NCI-ADR/RES^*∗*^	Ovary; adenocarcinoma	Ectoderm	26.40	>25.00
786-O	Kidney; adenocarcinoma	Mesoderm	28.00	0.038
NCI-H460	Lung; non-small-cell lung carcinoma	Mesoderm	27.50	<0.025
PC-3	Prostate; adenocarcinoma	Mesoderm	24.80	0.025
OVCAR-3	Ovary; adenocarcinoma	Mesoderm	4.40	0.230
HT-29	Colon; adenocarcinoma	Endoderm	7.90	0.026
K562	Bone marrow; chronic myeloid leukemia	Mesenchyme	>250	>25.00

Results are the mean ± SD of 3 replicates per treatment in three independent tests after 48 hours of treatment.

CE: crude extract of *Amphipterygium adstringens*.

DOX: doxorubicin (positive control).

^*∗*^Multidrug resistant cell line.
